# Does geography matter? Implications for future tourism research in light of COVID-19

**DOI:** 10.1007/s11192-022-04615-z

**Published:** 2023-01-11

**Authors:** Judit Sulyok, Beáta Fehérvölgyi, Tibor Csizmadia, Attila I. Katona, Zsolt T. Kosztyán

**Affiliations:** 1 Department of Tourism, Faculty of Business and Economics, Institute of Business, University of Pannonia, Hungary, Egyetem str. 10, Veszprém, 8200 Hungary; 2 Department of Management, Faculty of Business and Economics, Institute of Management, University of Pannonia, Egyetem str. 10, Veszprém, 8200 Hungary; 3 Department of Quantitative Methods, Faculty of Business and Economics, Institute of Management, University of Pannonia, Egyetem str. 10, Veszprém, 8200 Hungary

**Keywords:** Tourism, COVID-19, Review, Research methods, Spatial network, Text mining

## Abstract

Due to the 2019 new coronavirus disease (COVID-19) pandemic, tourism is undergoing fundamental changes that are affecting tourism research. This situation calls for in-depth analyses of tourism research. Scholars have already published review studies on COVID-19-related research within the tourism field; however, these studies do not connect findings, such as the research focus, research methodology and target group, to form a research profile, and the geographical patterns of the findings are not identified. study, COVID-19-related tourism studies were collected and analyzed in depth following the Preferred Reporting Items for systematic reviews and meta-analyses (PRISMA) method. In addition, data-driven methods, such as spatial multilayer networks, frequent patterns and content-based analyses, were applied to identify research profiles and their geographic patterns. This study pointed out the role of geographic patterns in tourism research, going beyond the research of the authors. Moreover, topics, focus destinations, applied methodologies and employed data sources have relevant geographic patterns. Four dominant research profiles that show that a shift can be observed in tourism research toward data sources and research methods were identified. Due to COVID-19, the strengthening of the application of quantitative methods and employment of secondary data sources are needed.

**Supplementary Information:**

The online version contains supplementary material available at 10.1007/s11192-022-04615-z.

## Introduction

Since its outbreak in 2019, the new coronavirus disease (COVID-19) pandemic has fundamentally affected the world. Economies and societies face significant challenges that have brought about increased attention to the rethinking of everyday life and business. Tourism is among the sectors most affected by the pandemic. Moreover, until 2019, sustainability was at the forefront of the minds of tourism stakeholders because of overtourism; now, the industry is facing a phenomenon of nontourism. Papers reporting the impacts of COVID-19 on various aspects of the tourism industry, such as physical, social and economic aspects, have begun and continue to emerge (Gössling et al., 2020; Hall et al., 2020; Michalkó et al., 2022; Ozili, 2022; Sigala et al., 2020; Uğur & Akbıyık, 2020). The epidemic indicates the change in research focus and specific methods such as in the absence of tourists (Pillai et al., 2021); it was not possible to conduct tourist-centric research in a tourist-centric sector where the economy-based paradigm dominates (Pillai et al., 2021), which is an issue that had been criticized before the pandemic by Adam Franklin (Franklin, 2018). As we move toward the phased unlocking and restarting of tourism, scholars should adopt a retrospective approach to reflect on how to build back better while also strengthening the resilient capabilities needed to survive in this highly turbulent and obscure global environment (Gretzel et al., 2020). Systematic literature reviews are invaluable scientific tools (Mulrow, 1994) that provide a comprehensive overview of the tourism research field (Le et al., 2019) and, due to the clearly stated procedure, enable the easy replication of results (Templier and Paré,^2018^).

Tourism development also impacts how the sector should or could be addressed in academic research (Korstanje & George, 2022). The implications of COVID-19 for tourism as a socioeconomic phenomenon and industry practice have fueled research in the tourism field (Utkarsh and Sigala, 2021), thus increasing the number of publications in this area (Sigala et al., 2020). Although publication growth is also valid for non-COVID-19 studies, the changing publication pattern included a significantly faster mean time to acceptance for COVID-19 papers (Aviv-Reuven & Rosenfeld, 2021). In addition, journals have developed special issues and/or are welcoming any form of academic paper from several locations addressing tourism issues caused by the pandemic. Furthermore, industry reports issued by national tourist organizations were also important sources of knowledge and input for numerous studies (Rogerson & Rogerson, 2021a, 2021b). Along with the boom in academic publications, the originality of research published has also become a cutting-edge issue: originality can be understood regarding the theory, method or setting used (Buckley, 2022).

For a better understanding of this phenomenon, tourism scholars have published review studies on COVID-19-related research within the tourism field. For example, Sharma et al. (2021) reviewed COVID-19-related research in tourism and focused on systematically reviewing research solely related to resilience and COVID-19 in tourism. Sigala et al. (2020) published a bibliometric review of the research on COVID-19 and tourism, and Zopiatis et al. (2021) conducted an integrative review of COVID-19 and the tourism field. Citation network analysis and frequency analysis were performed on abstracts and keywords but did not address the body of the selected academic papers. These analyses identified the most cited researchers and most frequent terms. Although all of these papers are valuable, their findings are not connected to each other to allow us to identify with whom, with what, and where to conduct research. In addition, the tourism sector has strong geographical embeddedness, and tourism mobility can be understood in space. Although COVID-19 has impacted the entire industry worldwide, such impacts might show geographical differences, such as cities experiencing a greater decline than rural areas, which can lead to growing polarization (Rogerson & Rogerson, 2022). Geographical location influences primarily which target group is the research focus. In this way, the research questions and, indirectly, the research hypotheses are influenced by geographical location. Therefore, in the case of review research, geographical aspects cannot be neglected (Fontana et al., 2019). Wu (2013), Ahlgren et al. (2013) pointed out that due to globalism, the average distance between citations and that between the locations of co-authors are increasing. At the same time, this collaboration is made difficult by epidemic situations (Aviv-Reuven & Rosenfeld, 2021; Yang et al., 2020). Moreover, tourism scholars have adopted various and divergent methodological approaches to investigate the multifaceted nature of COVID-19 and its implications for tourism research and industry. As this literature stream evolves and continues to attract research attention and contributions, the exploration of the profiles of the research that has already been conducted within the field of tourism and COVID-19 by mapping the methodological aspects of tourism papers is both overdue and important.

To fill this gap, this study adopts a systematic literature review to review the studies published in the field of tourism and COVID-19 based on a complete database (DB) of COVID-19-related scientific publications provided by Dimensions.ai. By revealing, analyzing and synthesizing COVID-19-related tourism research, we expand the literature by mapping the relationship between the methodological and geographical dimensions and by offering several important theoretical and practical implications. Specifically, this paper aims to

- $\hbox {A}_1$ explore the geographical patterns of tourism research;
- $\hbox {A}_2$ identify research profiles and their relations by mapping the methodological aspects of tourism research; and
- $\hbox {A}_3$ analyze the effect of COVID-19 on academic tourism research.

Relative to existing studies, the major contributions of this paper can be summarized as follows. First, this study explores the geographical patterns of tourism research that can lead to a deeper understanding of destination-specific attributes, in line with the sociocultural background of the addressed study areas. The approach involving the geographical patterns of tourism research is highly relevant, as it can lay the groundwork and help us identify opportunities upon which future research can be built and help improve international collaboration processes in the tourism field. Second, identifying and mapping research profiles provides scholars with a multidimensional tool for analyzing research in space. Analyzing research profiles provides scholars with the current progress of elements such as the research focus, research methodologies and target groups. Proposing a multilayer network analysis enables scholars to reveal the structure of the analyzed research field based on several perspectives, such as citation behavior, geographical patterns, methodological aspects, data sources used, and research content. Each layer treats a specific research component, and the proposed multilayer network structure allows scholars to analyze the relations between the identified research components. The structure of these research components (such as research methods, data sources, and target groups, each of which are in a different layer) provides research profiles, which can be country or regional specific. This approach can allow us to reflect on what, with whom, where and how to conduct research to contribute to better tourism in the future. Third, relying on the proposed methodology, this study provides insight into the inclusive landscape of tourism research in the context of COVID-19. This work can help us better understand and identify the shifts caused by COVID-19 in academic tourism research focus.

## Study background

Due to the COVID-19 pandemic, tourism is undergoing fundamental changes. Although the tourism phenomenon itself has been seriously impacted, the need for tourism-related academic publication has not stopped. In 2021, 10,752 referenced articles were published in 272 journals (McKercher & Dolnicar, 2022). The lack of tourists has been obvious during COVID-19, and the great amount of available digital information (e.g., Skyscanner, TripAdvisor, and Airbnb data) has enabled researchers to analyze demand (Casado-Aranda et al., 2021). Academic tourism knowledge is driven by leading authors, AD Scientific Index^1^ nd globally recognized institutions, Shanghai Ranking^2^. The most productive institutions play a fundamental role (Zhang et al., 2015). In addition to the dominance of the US, the UK and China (Roychowdhury et al., 2022), Australia plays a cutting-edge role (Rogerson & Rogerson, 2021a; Zhang et al., 2015) in disseminating scientific knowledge in the tourism field. This picture has not changed fundamentally since COVID-19 pandemic times, with leading tourism scholars providing guidance for tourism research in the future as authors of academic papers (Gössling et al., 2020) or guest editors of COVID-19-related special issues, such as Tourism Geographies 2020 vol. 22 issue 3.

On the one hand, the academic literature has aimed to address what the future of tourism may look like. On the other hand, researchers have called for a rethinking of tourism research (Schweinsberg et al., 2021). Moreover, various tourism studies have simply confirmed what is already known; thus, the new research should be forward looking (Kock et al., 2020), which could mean using new theories and models in tourism (such as the evolutionary tourism paradigm used by Kock et al. (2020)), or the joining of forces between tourism and other fields. Wen et al. (2021) calls for interdisciplinary studies in the tourism and health industries, i.e., integrating diverse perspectives that result in a better understanding. In this case, interdisciplinary means integrating knowledge/expertise but not providing methodological support. The importance of interdisciplinary studies is also emphasized by Liu et al. (2021).

However, there is no doubt that existing, well-developed research protocols must be implemented (Schweinsberg et al., 2021). The diverse perspectives of different stakeholders, such as service providers, employees, customers, and travelers, have been highlighted by (Zopiatis et al., 2021). The longstanding transformational impacts of COVID-19 also focus on how individuals’ mindsets change and how policy makers, including political and institutional stakeholders, act to achieve better tourism in the future (Hall et al., 2020).

Taking a retrospective look, evolutionary trends in tourism have an impact on academic research as well. For example, technological advances have resulted in an increase in digital content originating from both the demand and supply sides; thus, a great number of academic papers deal with content analysis (Stepchenkova, 2012). Alternatively, tourism mobility has been criticized because of sustainability issues, and various publications deal with sustainability (Casado-Aranda et al., 2021). Tools supporting tourism research are also reflected in academic papers: Casado-Aranda et al. (2021) using SciMAT software, Leong et al. (2020) and Utkarsh and Sigala (2021) using VoSViewer, etc. Regarding methodological advances in tourism, there are also studies in which authors apply certain methods in the hospitality sector, e.g., Kemperman (2021) discusses discrete choice experiments in tourism.

Much of the current literature on the effects of COVID-19 on tourism pays particular attention to the sector, namely, how the pandemic affects tourism itself (Uğur & Akbıyık, 2020), and less attention to tourism research. Even before the outbreak of the epidemic, Franklin (2018) criticized tourism research because it was too tourism-centric. In the absence of tourists, however, it can be expected that the opportunities for tourism research will have fundamentally changed during the epidemic. Academic discussions about tourism research were present before the COVID-19 pandemic. Franklin and Crang (2001) highlighted troubles with tourism studies, including that researchers often track a phenomenon that quickly changes, the centricity of tourists, and the economic oriented understanding of tourism. Instead of being an isolated area, tourism is a dimension of global social life (Franklin & Crang, 2001). The complexity and links with other external factors, such as the sociocultural environment, should also be reflected in academic research, similar to how Franklin (2007) calls for a more distributed and translated sense of tourism. The COVID-19 pandemic situation resulted in an exciting and challenging time for work on tourism (Duffy et al., 2021, p. 5). For tourism studies, this means widening the scope, addressing theoretical perspectives and new topics (Duffy et al., 2021). Regarding these topics, it is interesting to take a look back at cases of sustainability and technology, which are two areas that have become key issues and will most likely remain so in the future, even though it was seen as Utopist to talk about these subjects only a few decades ago (Duffy et al., 2021). Most of the tourism-oriented academic articles dealing with the impact of COVID-19 address topics to be researched (what to do); fewer papers give any suggestions regarding methodological issues (how to do). Topics are often defined by the authors Liu et al. (2021), Casado-Aranda et al. (2021), such as by using word counts or by following the theoretical framework of the tourism industry. Regarding future research directions, sustainability and smart tourism are at the forefront (Casado-Aranda et al., 2021). The topics covered by academic papers have been clustered by (Zopiatis et al., 2021) into the pandemic impact, relevant issues in the post-COVID-19 era, and the pandemic impact on tourist perceptions. Other relevant and forward-looking themes, such as the resilience of tourism (McKercher, 2021), second homes (Zoğal et al., 2020), sustainability or responsible tourism behavior (Stankov et al., 2020), have also appeared in academic papers. The concept of relevance concerns not only the areas affected by COVID-19 but also the underpinning of the potential of new research areas (Zoğal et al., 2020). New research focuses, often published as “viewpoint papers” may be validated by ongoing trends, so they certainly have a place in academic works. To address the effects of COVID-19 on tourism, academic articles also include dedicated primary field work supporting decision makers with short-term available, practical implications (Wojcieszak-Zbierska et al., 2020; Pappas, 2021). In the case of the COVID-19 pandemic, the need for prompt information has resulted in supporting materials and reports issued by international organizations such as the Organization for Economic Co-operation and Development (OECD), United Nations (UN) World Tourism Organization or European Travel Commission, as well as by policy makers and destination management organizations (DMOs). Moreover, the proliferation of research notes, preprints, and open access journals may be considered a positive development (Zopiatis et al., 2021), while publication “fever” results in some cases having weaker methodologies, the results or conclusions and may be considered a negative development (Zopiatis et al., 2021).

From a methodological point of view, various types of mapping and synthesizing analyses on the existing body of knowledge have appeared in the tourism literature (Lim & Ok, 2021; Li et al., 2018). Among the different types of analyses conducted by scholars, systematic reviews have emerged as one of the main strategies to assess the status of tourism knowledge (Yang et al., 2017; Le et al., 2019). In line with this, Pahlevan-Sharif et al. (2019) stated that employing transparent and comprehensive guidelines is important for minimizing bias and producing trustworthy assessments of the existing body of knowledge. According to the above authors, the **P**referred **R**eporting **I**tems for **S**ystematic Reviews and **M**eta-**A**nalyses (PRISMA) method can be employed by tourism scholars, as it represents one of the most comprehensive processes for planning, preparing and publishing systematic reviews.

In tourism research, literature reviews often use the PRISMA data collection method with descriptive analyses (Oviedo-García, 2016; Yang et al., 2017). In addition, some papers use co-occurrence networks, citation networks and coauthorship analyses (provided by VOSviewer) (Jiménez-García et al., 2020; Leong et al., 2020) or bibliographic coupling (Zopiatis et al., 2021). Weismayer and Pezenka (2017) applied the latent semantic analysis of keywords to reveal the emerging fields in tourism. A limited number of studies have dealt with the geographical patterns in tourism-related literature reviews (Cavalcante et al., 2021; Rosalina et al., 2021; Roychowdhury et al., 2022; Zhang et al., 2015). Only a few of these works have taken additional steps to analyze the geographical patterns according to their spatial scopes of interest (Cronjé & du Plessis, 2020; Demiroglu & Hall, 2020). Furthermore, only one paper has classified studies in terms of methodological aspects (Wut et al., 2021), but it has not dealt with geographic patterns related to the topics of interest.

Tourism research is inseparable from examined places. Therefore, geographical patterns should be sought not only in citation networks (as suggested by Ahlgren et al., 2013; Pan et al., 2012; Wu, 2013) or in topics (Fontana et al., 2019) but also in the whole layer of the research, such as focus destinations and data sources or even in the methodologies. Topics and focus destinations may determine the employable data sources. Data sources may determine which kind of methodology can be used. Therefore, the proposed research profiles should be examined instead of exploring only one aspect.

In this paper, we perform a systematic review of the extant tourism research focusing on the various aspects of COVID-19 by combining several quantitative approaches, such as topic modeling, geospatial multilayer network analysis (including geospatial citation networks and focus destination networks) and frequency pattern analysis. Our paper specifies geographically related research profiles, considering the data source, content, methodology, and target group.

The above discussion drives our motivation to perform a review of the challenges faced by the global tourism industry affected by COVID-19. The research questions for our study are set as follows:

- $\hbox {RQ}_1$ What geographical patterns of tourism research can be identified?
- $\hbox {RQ}_2$ Can research profiles and their relations be identified by mapping the methodological aspects of tourism research?
- $\hbox {RQ}_3$ What effects of COVID-19 on academic tourism research can be identified?

## Research methods

The main purposes of this research are to analyze the effect of COVID-19 on academic tourism research ($\hbox {A}_3$). To *identify research profiles* in tourism in the COVID-19 era ($\hbox {A}_2$) and to *analyze their geographical aspects* ($\hbox {A}_1$), first, the components of research profiles are identified, and their relationships are subsequently analyzed.

In this study, a data-driven approach is employed to analyze the geographical aspects of tourism research profiles in the COVID-19 era. In contrast to the traditional model-driven approaches, this approach could not be based on a preliminary research model and the associated research hypotheses. However, clearly defined research purposes and associated research questions have been formulated.

To meet these purposes, first, a set of relevant papers were selected by employing the most widely used systematic literature review technique (PRISMA). Following the combination of text mining and deep review efforts, components of research profiles were identified. Then, all components were geo-coded, and the patterns of the relationship between components of research profiles were identified.

The outcome of the research process was a network of the relationships between the components of the research profiles. This network could be considered to be a model for future research, which can be tested by model-driven methods in any regional study.

### Data collection

In this section, the data collection process and the applied data tables are introduced. The analysis conducted in this paper was based on the DB of COVID-19-related scientific publications provided by Dimensions.ai.

**Fig. 1:**
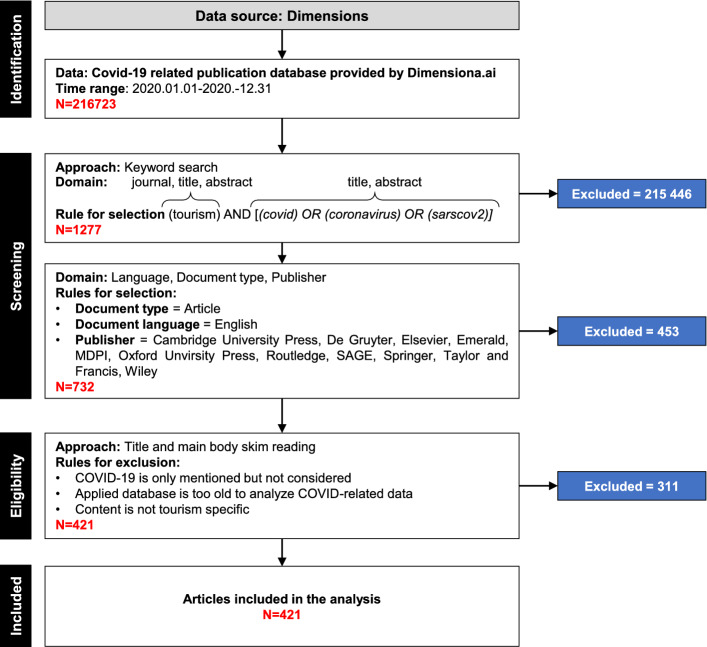
PRISMA flowchart representing the data collection and preparation process

During data collection and further preprocessing, the PRISMA methodology proposed by Moher et al. (2010) was applied, as it guides researchers in conducting a systematic literature review and consists of four fundamental steps: (1) identification, (2) screening, (3) eligibility and (4) inclusion. Figure 1 shows the data collection and preprocessing steps applied. The excluded number of papers is also highlighted at each step.

(1) Identification: The DB provided by Dimensions.ai was applied as the basis of the analysis since it is a comprehensive DB with hundreds of thousands of records. At the time of download, the DB covered all of 2020, including 216,723 records of scientific publications with overall COVID-19-related content.

(2) Screening: In this case, the removal of duplicated records was not necessary since only one large DB was applied during data collection. Data screening could be separated into two phases.

First, tourism-related publications were detected by a keyword search. The search was applied considering the title, abstract and journal name using the keyword *tourism*. At this point, it is important to note that this study was not limited to tourism-related journals. In the keyword search rule, the journal name was included with an “*OR*” operator to avoid missing relevant papers published in nontourism-related journals .

As a refinement, a further keyword search was conducted on titles and abstracts with COVID-19-related terms such as *COVID*, *coronavirus*, and *sarscov2*. The goal of this refinement was to ensure that only COVID-19-related publications were included in the analysis. At this step, the DB was reduced to 1277 records by excluding 215,466 records based on the aforementioned criteria.

In the second phase of screening, the records were further filtered by language, document type and publisher. Only English-language publications were kept in the DB. There are two reasons for considering only English-language papers in the study. First, this study follows the language inclusion rule to ensure the consideration of international, peer-reviewed and high-quality papers. Second, in this paper, the application of text mining approaches plays a fundamental role; therefore, including documents in multiple languages could strongly distort the results.

Regarding document type, only scientific articles were considered, and 11 highly prestigious publishers were selected for paper filtering, ensuring that the analyzed publications were peer-reviewed, high-quality papers. After the application of these filtering criteria, 732 scientific papers remained in the DB after excluding 453 papers compared to the previous state.

(3) Eligibility: In this part, manual reading was applied to the remaining 732 papers. On the one hand, they were investigated from perspective of relevancy. On the other hand, important information, such as that below, was extracted during the reading:

- Type of data source used (primary, secondary, combined or none);
- Type of research method used (quantitative, qualitative, mixed, literature review or none);
- Target groups as the focus of the analysis (service providers, policy makers, tourists or residents);
- Geographical area analyzed as the focus destination (at the country level); and
- Formulated research questions and research hypotheses.

At this step, 311 additional papers were excluded because they were not relevant for the following reasons: (1) they mentioned COVID-19 but did not analyze it, (2) the applied DB in the paper was too old to investigate COVID-19-related data, or (3) tourism was mentioned, but the content was not tourism specific.

(4) Inclusion: After following the aforementioned steps, 421 relevant scientific papers remained in the DB. In the following section, we refer to the collected and preprocessed DB as the *COVID-19-Tourism DB*.

### DB structure

In the following section, the different tables used in the analysis are introduced. In this paper, five different data tables were constructed, according to the following structure, as part of the *COVID-19-Tourism DB*:

- Data table resulting from data collection and preprocessing (1 table);
- Network-related data tables (4 tables);
  - Node and edge tables representing the citation network (2 tables); and
  - Node and edge tables representing the focus destination network (2 tables).

First, the data table resulting from data collection and preprocessing is presented, with Table 1 providing a description of the different columns.

**Table 1:** Specification of the data table resulting from data collection

Group	Nr.	Column name	Description
Paper	1	Digital object identifier (DOI)	DOI of the given paper, which is used for the identification of papers
	2	Title	Title of papers
	3	Abstract	Abstract of papers
	4	Text	A concatenated field of Title and Abstract, which is used for text analytics
Focus destination	5	Dest_type	Destination type such as country or region
	6	Dest_focus_region	Region of the destination of focus
	7	Dest_focus_country	Country of the destination of focus
	8	Dest_focus_subregion	Subregion of the destination of focus.
Research scope	9	Target_group	Target group of the analysis such as tourists, policy makers or residents
	10	Data_source	Type of data source used
	11	Research_method	Research method used
	12	RQ_RH	Research question and research hypothesis stated in the paper
Affiliation data	13	Affiliation_continent	Continent of the first author’s affiliation
	14	Affiliation_country	Country of the first author’s affiliation
	15	Affiliation_ISO3	ISO3 code of the first author’s affiliation

The data table resulting from the data collection process includes 421 records (as a result of the PRISMA method) and 15 columns. The columns can be assigned to four main groups based on their information content: (1) columns with paper-related information, (2) columns associated with the focus destination area, (3) columns describing the research scope of the papers and (4) columns with affiliation-related data. This data table was used to conduct text mining and geographical analyses in this paper.

To conduct network analysis, node and edge tables also need to be constructed. Table 2 represents the structure of the node tables, where the “Network” column denotes the given network type (the node table associated with the given network).

**Table 2:** Specification of node tables used in network analysis

Network	Column number	Column name	Description	Data type
Citation	1	ID	ID of the given paper, which is used to refer to the paper in the edge table	Integer
	2	Label	A short label of the paper containing author and publication year data	String
	3	Latitude	Latitude related to the affiliation of the first author	Double
	4	Longitude	Longitude related to the affiliation of the first author	Double
	5	DOI	DOI number of the papers	String
	6	ISO3	ISO3 code of the country related to the first author’s affiliation	String
	7	TC	Total number of citations given by the Dimensions DB	Integer
Focus destination	1	ISO3	ISO3 code of the paper’s country or the destination country of focus	String
	2	Type	Type of the node: its value is either “paper” or “focus”, indicating whether the node refers to a paper or a destination area of focus, respectively	String
	3	Latitude	Latitude of the paper’s affiliation (first author) or the latitude related to the geographical center of the destination country of focus	Double
	4	Longitude	Longitude of the paper’s affiliation (first author) or the longitude related to the geographical center of the destination country of focus	Double

In the case of a citation network node table (the upper part of Table 2), each record represents a paper. Not only paper-related information but also geographical variables were included, such as latitude, longitude and ISO3 country code. The latitude and longitude values were specified by using the

Python *GeoPy* package , which is able to retrieve latitude and longitude values for a given geographical object such as a city or an institute. To determine the geographical coordinates, the first author’s affiliation (institute) was used as the input parameter.

In the node table associated with the focus destination network (the lower part of Table 2), each record represents a country. Similar to the citation network node table, geographical variables were determined by using GeoPy. It is necessary to note that in this network, there are two types of nodes; i.e., they can refer to the paper’s country or to the country of the destination area of focus. In the case of a paper-type node, the coordinates point to the center of the country based on the first author’s affiliation. If the node type is “focus”, then the coordinates refer to the center point if the country is related to the destination area of focus.

The example records for the node tables used in the citation network analysis and focus destination network analysis are formulated as follows:

Node table example record used in the citation network analysis:

$$\begin{aligned} {[}1 |\hbox { wen (2021) }|\hbox { -31.9193 }|\hbox { 115.8691 }|\hbox { 10.1108/tr-03-2020-0110 }|\hbox { AUS }|\hbox { 72}] \end{aligned}$$

Node table example record used in the focus destination network analysis:

$$\begin{aligned} {[}\hbox {ITA }|\hbox { focus }|\hbox { 41.00989 }|\hbox { 28.95977}] \end{aligned}$$

Edge tables are also required to conduct network analysis, and those associated with the two types of networks are described in Table 3.

**Table 3:** Specification of the edge tables used in network analysis

Network	Column number	Column name	Description	Data type
Citation	1	From	ID of the citing paper	Integer
	2	To	ID if cited paper	Integer
	3	Country_from	ISO3 code of the citing paper’s country, which is determined based on the first author’s affiliation	String
	4	Country_to	ISO3 code of the cited paper’s country, which is determined based on the first author’s affiliation	String
Focus destination	1	ISO3_from	ISO3 code of countries where papers were published (first author’s affiliation)	String
	2	ISO3_to	ISO3 code of countries as the destination of focus of the papers	String
	3	Papers	Number of papers that were written in the country defined by “ISO3_from” and focusing on the destination country defined by “ISO3_to”	Integer

To build an edge table based on citation data, the citing references were determined by the *Dimensions* platform. The DOI numbers of the 421 papers were uploaded to the system where the cited references were exported. After this step, *VOSviewer* was used to build the edge table for the citation network at the paper level. In the case of the focus destination network, there was no need to use further queries or software since the paper location-focus destination location pairs were determined as part of the PRISMA method in step 3 (see Subsection Data collection). The example records for the edge tables used in the citation network analysis and focus destination network analysis are formulated as follows:

Edge table example record in the citation network analysis:

$$\begin{aligned} {[}\hbox {3 }|\hbox { 15 }|\hbox { AUS }|\hbox { POL}] \end{aligned}$$

Edge table example record in the focus destination network analysis:

$$\begin{aligned} {[}\hbox {GBR }|\hbox { US }|\hbox { 3}] \end{aligned}$$

### Methods employed

In our study, *data-driven methods* were mainly applied. First, both for the citation and focus destination networks, *spatial networks* were employed (see Section Spatial networks), where the location of the author or the focus destination was the primary property. On the proposed spatial networks, modules were specified through community-based analysis, which provided a set of nodes (i.e., modules), where the nodes within a module were more densely connected than were the nodes between two distinct modules (see Section Community-based analysis). Since community-based analysis is a spatially invariant method, the set of geographically connected countries in a module indicates regional groups with similar research interests.

**Fig. 2:**
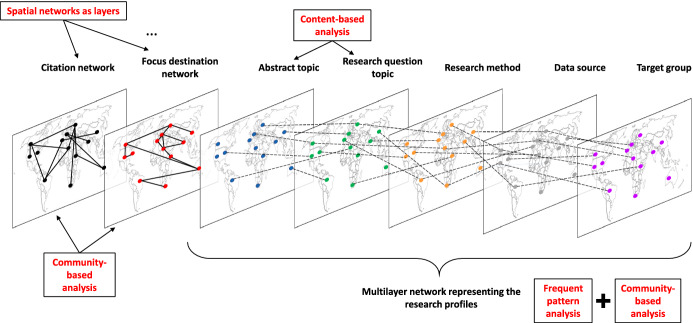
Analysis framework

Different spatial networks with the same nodes (i.e., countries) provide a set of networks, or in other words, a multilayer network (see Fig. 2). The frequent edges between nodes (see the interconnections in Fig. 2) from different layers indicate the similarity of research in terms of data sources, research methods, target groups and focus destinations (see Section Frequent pattern analysis (FPA)).

As long as data sources, resource methods or target groups can be categorized manually, when categorizing abstracts, research questions and hypotheses, text mining techniques, such as content-based analysis, must be applied (see Section Content-based analysis).

#### Spatial networks

A network can be described as a tuple $G=(V, E)$, where *V* denotes the set of nodes, and *E* denotes the list of edges in the network. Furthermore, $e_{ij}$ denotes the linkage between nodes $v_{i}$ and $v_{j}$ $(i,j \in {1,...,n})$, and $w(e_{ij})$ is the weight of the edge between nodes $v_{i}$ and $v_{j}$. Spatial networks are special network cases, where the nodes and edges are embedded in space and have spatial characteristics such as geocodes (latitude and longitude values) in the case of nodes and distance metrics in the case of edges, such as spatial distance or social distance (Barthélemy, 2003, 2011).

Spatial networks are organized as a multilayer network, which is a pair, $\mathcal {M}=(\mathcal {G},\mathcal {C})$, where $\mathcal {G}=\{G_{\alpha }=(V_{\alpha },E_{\alpha }),\alpha \in \{1,..,m\}\}$ is a family of (directed or undirected and weighted or unweighted) graphs (called layers of $\mathcal {M}$), where $V_{\alpha }$ is the set of vertices (set of nodes), and $E_{\alpha }$ $\subseteq V_{\alpha }\times V_{\alpha }$ is the set of edges (links) of graph $G_{\alpha }$ in layer $\alpha$. Moreover:

1 $$\begin{aligned} \mathcal {C}=\{E_{\alpha ,\beta }\subseteq V_{\alpha } \times V_{\beta },\alpha ,\beta \in \{1,..,m\}, \alpha \ne \beta \} \end{aligned}$$

is the set of interconnections between nodes of different layers $G_{\alpha }, G_{\beta }\in \mathcal {M}$ with $\alpha \ne \beta$.

The set of nodes are countries in all networks. Frequent edges between nodes (i.e., countries) provide a community within a layer (i.e., spatial network) but also specify a new network between categories of distinct networks (see the interconnections in Fig. 10).

#### Community-based analysis

Within a noninterconnected layer or a distinct network, the goal of community-based analysis is to find a good partition, $V = {C_1, C_2,..., C_m}$, of the analyzed network *G*, where *m* is the number of communities found over *G*, and each $C_i \subseteq V$ is a set of nodes. The goodness of partitioning is often measured with the following modularity metric (Ghosh et al., 2018):

2 $$\begin{aligned} Q=\frac{1}{2w}\sum _{i,j}(M_{ij}-\frac{k_ik_j}{2w})\delta (C_i,C_j) \end{aligned}$$

where *w* is the sum of all edge weights, $M_{ij}$ is the adjacency matrix, $k_i$ is the weighted degree of node $v_i$, $C_i$ is the community of $v_i$, and $\delta (C_i,C_j)=1$ if $C_i=C_j$ and 0 otherwise.

*Q* reflects the difference between the fraction of edges within the communities compared to the so-called null model, which represents a random graph with identical node and degree distributions. The partitioning algorithm organizes the nodes into communities to maximize the modularity metric. In this paper, the Leiden (Traag et al., 2019) algorithm was used to find the best partition.

Gadar et al. (2018) pointed out that if a spatially invariant community-based detection method is used in a spatial network, then we can regain the connected spatial regions, which indicate regional communities.

To organize the network layout by visualizing the communities over the network, the *ForceAtlas2* layout algorithm was used since force-directed algorithms provide a good representation of the related clustering, as pointed out by Noack (2009). This algorithm establishes a force-directed layout, which simulates a physical system, where the nodes repulse each other similarly to charged particles and the edges attract their nodes as springs (Jacomy et al., 2014).

#### Frequent pattern analysis (FPA)

Frequent patterns are item sets that appear in a given dataset more times than a user-specified threshold frequency. Let $I = \{i_1, i_2, ... , i_n\}$ represent the set of all items in the dataset. An $\alpha$ itemset of *k* items is frequent if it occurs in the dataset no fewer than $\theta |D|$ times, where $\theta |D|$ is the total number of records of the *D* dataset. In this paper, the similarity between different research characteristics (item sets) is also measured to identify frequent patterns. For this purpose, the Jaccard similarity is used as follows:

3 $$\begin{aligned} J(A,B)=\frac{|A \cap B|}{|A \cup B|} \end{aligned}$$

where *A* and *B* are item sets, and *J*(*A*, *B*) denotes the Jaccard similarity between them.

Gadar et al. (2018) showed that FPA provides a new network on a noninterconnected multilayer network, where the nodes are the categories or modules of nodes, while the weight of the edges is the relative frequency of common nodes.

#### Content-based analysis

To perform a content-based analysis—latent Dirichlet allocation (LDA)—a topic modeling approach was used on textual fields such as the abstracts or formulated research questions/research hypotheses of the papers.

During the topic modeling process, LDA takes two distribution types into account: the document distribution over hidden topics and the distribution of words within these topics. Let *D* be the number of documents (papers) and *T* be the number of expected topics. The topic generation process can be described as follows (Jelodar et al., 2019):

1. For each $t (t\in \{1,...,T\})$, select a $\overrightarrow{\varphi }_t \sim Dir(\beta )$ distribution for the words
2. For each $d (d\in \{1,...,D\})$, select a $\overrightarrow{\theta _d} \sim Dir(\alpha )$ distribution for the topics
3. For each word $w(w\in \{1,...,N_d\})$ in each document *d*,
  - Choose a topic $z_n$ from $Multinomial(\overrightarrow{\theta }_d)$
  - Choose a word $w_n$ from $Multinomial(\overrightarrow{\varphi }_{zn})$

In the process, $N_d$ is the number of words contained by document *d*, and $Dir(\alpha )$ and $Dir(\beta )$ are Dirichlet distributions with parameters $\alpha$ and $\beta$, respectively; furthermore, $\theta$ and $\varphi$ denote multinomial Dirichlet distributions. *T*, $\alpha$, and $\beta$ are the hyperparameters of the model that must be specified.

## Results and discussion

### Descriptive statistics

Using the COVID-19-Tourism DB, several descriptive statistics are provided to characterize the scientific papers related to the COVID-19 context in tourism research. Table 4 shows the distribution of continents across the DB with the number of articles and dominant countries in each DB.

**Table 4:** Descriptive statistics of the articles

Continent	No. of articles	No. of countries	Dominant countries
Europe	175	32	UK (31)
Asia	137	21	China (48)
Americas	76	9	US (53)
Australia & Oceania	32	3	Australia (19)
Africa	20	11	South Africa (7)

Although COVID-19 has seriously impacted all tourism destinations in the world, there are imbalances regarding academic publications. In line with international tourism flows, most of the articles included in this study originate in Europe (175 articles, with 710 million international tourist arrivals in 2019) and Asia and the Pacific (169 articles, with 348 million tourist arrivals), followed by the Americas (76 articles, with 216 million tourist arrivals). Regarding leading countries within continents, the roles of the US (53 articles), China (48 articles), the UK (31 articles), and Australia (19 articles) are significant. The fragmentation of the analyzed academic works is also in line with international tourism performance. Within Europe, which is still the leading region in international tourism, 32 countries were addressed by studies. Ultimately, from the African continent, 11 countries were included in the articles, while only two countries from South America were covered by tourism-oriented papers discussing relevant COVID-19 issues.

Not only countries but also top publishers were analyzed. Table 5 shows the top publishers based on the total number of citations.

**Table 5:** Top publishers by total number of citations

Publisher	Total citations	No. of papers
Taylor & Francis	1,544	135
Elsevier	837	108
MDPI	270	89
Springer Nature	137	27
SAGE Publications	140	23
Emerald	305	19
Wiley	50	12
De Gruyter	3	3
Oxford University Press (OUP)	71	3
Cambridge University Press (CUP)	2	2

Although the most significant English-language publishers were included during the research process, it is worth taking a closer look at the ranking of the research sample. Two leading publishers—Taylor & Francis and Elsevier—generated 6 out of 10 papers, followed by Emerald and MDPI. The number of citations showed a slightly different picture; Taylor & Francis “took” 46 percent of the citations, followed by Elsevier (25 percent), Emerald (9 percent), and MDPI (8 percent). The citations of the selected articles are influenced by short-term factors, as publishing academic work may require a longer time than the researched period. Another influencing factor may be access to academic sources and subscriptions to publishers’ services. Here, it should be noted that, recently, an increasing amount of academic work has become available via open access (open access journals or open access articles).

Similar to the top publishers, the top journals were extracted from the COVID-19-Tourism DB. Table 6 presents the ten most-cited journals. Total citations are calculated as the sum of all paper citations within the given journal in the analyzed timeframe. The H index for each journal was extracted using the Scimago DB.

Table 6 shows the top 10 most-cited journals.

**Table 6:** Top 10 cited journals

Journal	Publisher	Citations	Papers	H index
Tourism Geographies	Taylor & Francis	817	30	61
Annals of Tourism Research	Elsevier	343	26	171
Current Issues in Tourism	Taylor & Francis	315	27	74
Sustainability	MDPI	155	50	85
Journal of Sustainable Tourism	Taylor & Francis	123	17	103
International Journal of Contemporary Hospitality Management	Emerald	119	5	86
Journal of Business Research	Elsevier	117	1	195
Tourism Recreation Research	Taylor & Francis	114	7	44
Journal of Tourism Futures	Emerald	98	6	15
International Journal of Environmental Research and Public Health	MDPI	84	11	113

The top journals in which the selected articles were published are dominated by tourism-oriented media. However, the transdisciplinary nature of tourism is demonstrated by the fact that two of the top 10 most-cited journals, namely, Journal of Business Research and International Journal of Environmental Research and Public Health, are not tourism specific. The number of papers and citations show some differences across journals, which highlights the impacts of accessibility/visibility and reputation. Moreover, Sustainability, Tourism Geographies, and Current Issues in Tourism have the highest number of works in the DB, which ranks Tourism Geographies in first place in terms of citations, followed by Annals of Tourism Research and Current Issues of Tourism. In addition to dedicating space for COVID-19-related articles, some journals have also published special issues on the subject; furthermore, recently, the publishing process time has been shortened/accelerated in some cases.

The top five higher education institutions (HEIs) are shown in Table 7. Total citations are aggregated as the sum of citations of papers in which the first author’s affiliation is the given HEI.

**Table 7:** Top five cited HEIs

HEI	Total citations	No. of articles	Location
University of South Australia	230	3	Australia
Edith Cowan University	172	4	Australia
University of Canterbury	123	3	New Zealand
Temple University	107	1	US
Copenhagen Business School	102	2	Denmark

Supporting home institutions also play a role in the resulting high-quality academic work. One-fifth of the citations of the analyzed articles were generated by the top five HEIs, three of which (University of South Australia, University of Canterbury, and Temple University) are among the top 50 HEIs ranked by ShanghaiRanking on the subject of hospitality and tourism management. Only one HEI (Copenhagen Business School) is not located in a country where English is the official language.

During the screening process, we also identified the characteristics of the relevant papers based on the explored dimensions, such as the applied data source, research method used and target group analyzed. Table 8 shows the frequency of papers based on these dimensions.

The applied data source of the selected articles refers to the source of data collected by scholars or others. Four main categories were identified: (1) primary (for example, surveys such as online surveys conducted through social media platforms, (online) questionnaires, participant observations and (in-depth) interviews), (2) secondary (for example, regular open access governmental or organizational data sources, such as the World Bank’s DB, Johns Hopkins University Center DB, population registers, and statistical office DBs; sources from the internet such as websites and the GitHub repository; Internet of Things (IoT) data sources such as smartphone positioning data or FlightRadar24 data), (3) combined, which involved the joint use of primary and secondary data sources, and (4) no category, where the authors did not use data sources (for example, they used theoretical models or scenario analysis).

The research methods used in the selected articles were divided into the following categories: (1) quantitative (for example, descriptive statistics, model-driven methods such as exploratory factor analysis, analysis of variance (ANOVA) tests, correlation analysis, and regression analysis), (2) qualitative (for example, exploratory ethnographic study, (multiple) case studies, and Delphi studies), (3) mixed methods, i.e., using both quantitative and qualitative methods, (4) literature reviews summarizing the previous research on a topic, and 5) no category (for example, historical descriptions).

The target groups of the selected articles were identified in line with the tourism theoretical framework. The supply and demand sides were separated; furthermore, the public and for-profit business organizations were mapped. The four main categories identified were (1) service providers (for-profit companies, e.g., hotels, restaurants, attractions, and travel agencies/tour operators), (2) policy makers (DMOs, local/regional public bodies, and HEIs), (3) tourists (including domestic and international, excursionists and overnight visitors and, in both cases, nonresidents), and (4) residents/local communities.

Table 8 characterizes the research scope of the analyzed papers within the collected DB considering the type of data source used, research method applied and target group(s) analyzed. This categorization was conducted during data collection, as described in Subsection Data collection. It is necessary to note that the sum of the frequencies within the “Target groups” section is not equal to the total number of papers since each paper can focus on multiple target groups at once.

**Table 8:** Frequency of the research scope of the papers

Dimension	Category	Frequency
Data source	Secondary	213
	Primary	152
	Combined	13
	None (neither of them)	43
Target groups	Service providers	222
	Policy makers	198
	Tourists	168
	Residents	15
Research method	Quantitative	210
	Qualitative	102
	Mixed	26
	Literature review	22
	None (neither of them)	61

Data availability during the pandemic has affected the input for the analyzed articles. Parallel with the lack of gathering primary data from tourists, the pandemic has resulted in space for methodological “innovation”, which means that researchers could take advantage of using existing statistical figures to track trends visible during recent decades and thus provide input for a new future after COVID-19. At the same time, accessibility to certain data has enabled calculations with existing mathematical-statistical models, which had not often been used previously. At this point, it must be highlighted that the development level of statistical data collection systems and accessibility for tourism researchers also influenced the potential of publication for more developed areas. The absence of visitors put the concept of the digital footprint into the forefront of the minds of tourism academics; several papers analyzing content shared by travelers or clientele (e.g., in the case of restaurant services) exist. Regarding service providers and policy makers (as indicated above), primary research was the most popular mode to obtain insights into the impacts of the pandemic and about the solution that could potentially ensure the “survival” of business units. The need for “quick” results concerning the impact of COVID-19 on tourism was definitely a driver behind the publication of the analyzed articles, although tourism policy makers have not been the primary target group of academic works; open access publications are easy to access for nonacademic actors. The pandemic also presented an occasion to systematically analyze the available data gathered during recent years or even decades. The transdisciplinary nature of tourism calls for a wider understanding and more complex research method, which was only partially supported by this study and is in line with the general tendencies of academic publishing. Moreover, many tourism researchers have performed comprehensive research work, including qualitative and quantitative data collection. In the case of a high-quality publication, its narrow focus results in a low degree of mixed methods being used.

Regarding target groups, the categorization allowed us to indicate more segments; on average, 1.4 targets per paper were included in the DB. Most of the selected academic papers addressed service providers, followed by policy makers. These two groups were highly affected by COVID-19, and academic research also played a role in seeking solutions for the “survival” of companies and institutions. With the cessation of tourism flows caused by the pandemic, the core of the hospitality sector, namely, the travelers themselves, received less attention. Tourists were not able to be reached, and tourism consumption was taken over by other sectors. Beyond the pandemic, overtourism, or the undermanagement of tourism, has become a high-priority issue, putting local communities at the forefront of academic work; however, only a small number of COVID-19-related articles have dealt with local residents.

### Resource map

**Fig. 3:**
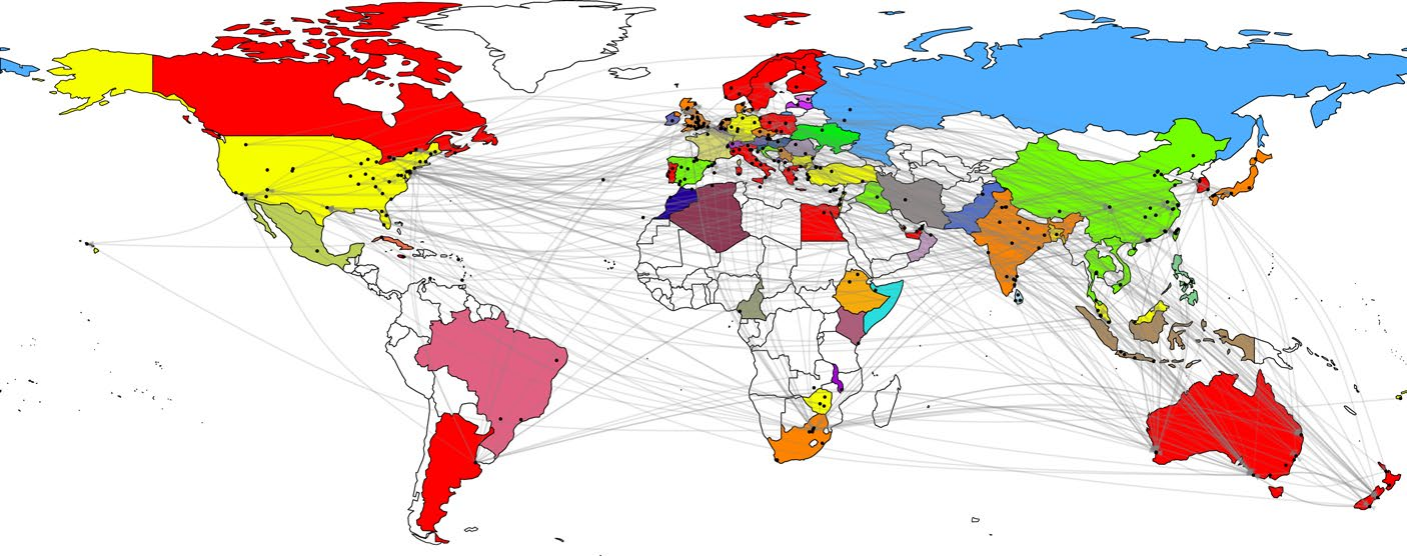
Citation network of COVID-19-related articles in tourism

Figure 3 shows the citation network of COVID-19-related articles in the field of tourism. Leiden’s community-based modularity detection algorithm clustered the citation network. The same colors indicate a common module, a node represents the location of the first author’s first affiliation, and an arc between nodes represents a citation for a paper from the given COVID-19-Tourism DB.

Although the analyzed DB included early publications linked to the effects of COVID-19 on tourism, some of the papers, which were written by leading tourism researchers, such as Hall et al. (2020) (with 120 citations), Sigala et al. (2020) (with 117 citations), Yang et al. (2020) (with 107 citations), and Higgins-Desbiolles (2020) (with 100 citations), achieved a significant number of citations during this short period. The academic works of these authors also serve as a fundamental guide regarding COVID-19 tourism impacts. Although the citation network was quite “colorful”, there were some leading groups regarding cocitations that showed an academic link between these areas. These groups did not follow a continent-based approach, as they included locations in different areas. The largest community included leading academic places such as Australia and New Zealand, as well as Scandinavian countries, Canada, Poland, Italy, Portugal, Greece, Egypt, South Korea, the United Arab Emirates, Jamaica, and Argentina. Regarding the citation network, the US was in the same group as Germany, Turkey, Malaysia, Fiji, Israel, Lebanon, Zimbabwe, Grenada, and Bulgaria. Not surprisingly, the UK had a citation link with India, but Japan, the Netherlands, Serbia, South Africa, Denmark, the Czech Republic, Qatar, and Singapore were also found in this community. The last “large” region included Spain, Cyprus, China, Croatia, Thailand, Taiwan, Vietnam, and Iraq as a source for citations.

Although the observed phenomenon is distance dependent, tourism-oriented academic studies show a distance-independent structure regarding citations, which is indicated by the spatially separated communities of countries.

**Fig. 4:**
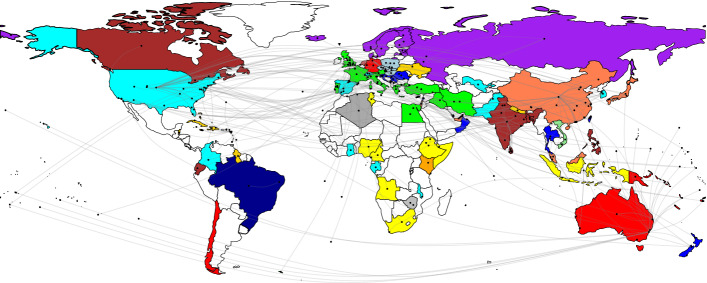
Focus destination network

Figure 4 shows the destination in the focus network. The source node shows the location of the first author’s affiliation, and the target node shows the country of the destination of the research. After Leiden’s community-based modularity detection, the common colors show the same modules.

The analysis concerning the destination of focus enables us to map links between places’ role in international tourism and in COVID-19-related academic papers. The top destinations studied in academic papers are those where COVID-19 severely impacted the tourism industry. Another reason for this may be that some of these countries, such as China, the US or the UK, are leading areas in academic publications at the same time. Among the top destinations and source markets in international tourism (UNWTO, 2019), China (38 items) and the US (29 items) had the most papers dealing with this topic in the DB, followed by Spain (21 items), Italy (19 items), South Korea (10 items), France (9 items), and the UK (9 items). In the case of China, in addition to Chinese authors, the US and the UK generated more papers that highlighted the importance of tourism flows between the two countries. The US was the focus of authors originating in China, the UK, and smaller European countries, e.g., Denmark, Poland and Portugal. Out of the top ten source markets in international tourism, Canada was focused on only one of the analyzed academic papers (Canadian authorship).

Considering the link between the first author’s affiliation (country) and the destination of focus, some interesting issues emerge as outcomes. Most of the countries—e.g., Brazil, Finland, Greece, Malta, and Romania—focus on their own destination, probably in line with the role played by HEIs in their environment as providing knowledge and because of the experience and availability of the necessary input for research. Another research area observed in the DB involves those papers that deal with important source markets (e.g., the UK and the US in the case of an author from Cyprus; France and Germany in the case of an author from Portugal; or Australia, China, and the US in the case of an author from the United Arab Emirates). Authors from the UK and the US focused on various destinations within the DB, possibly due to the role/knowledge of HEIs/researchers as academics.

In the case of Australian, African (from Cameroon), Czech, Latvian, Norwegian, and Serbian authors, there was an attempt to address neighboring/regional countries in the research. In this way, similarities across island destinations close to Australia, African countries, Northern Europe (Scandinavia and the Baltic region), 4 Visegrád countries, or the Balkan area result in the increased attention of tourism professionals being paid to a wider area.

Going a step further, the colors on the map show areas where there is an interest among countries in dealing with each other, rather than focusing on places not belonging to the same group. Australian tourism researchers have covered neighboring islands in their studies. In the case of COVID-19-related research, the Mediterranean region includes Eastern and North African regions. Interestingly, the UK belongs to this group, which means that UK authors are focusing on these areas as well. However, Spain is not part of this group; it has a stronger link with the US, Latin America, and some African and Asian countries. Within Asia, two main groups could be differentiated, with India and China in the lead. The African continent is dominated by one segment; however, New Zealand has a wide range of links within this group (e.g., Croatia, Oman, and Thailand). Northern Europe is also one region, including Scandinavia, the Russian Federation, and the Baltic countries. Moreover, Caribbean destinations and 4 Visegrảd countries in Central Eastern Europe also form separate groups. Furthermore, some of the countries in the COVID-19-Tourism DB have no link with other areas: Malta, Brazil, Kenya, Vietnam, and Algeria. The COVID-19 pandemic has fundamentally reshaped both the world and the tourism industry. However, this change is not reflected in the related academic studies.

**Fig. 5:**
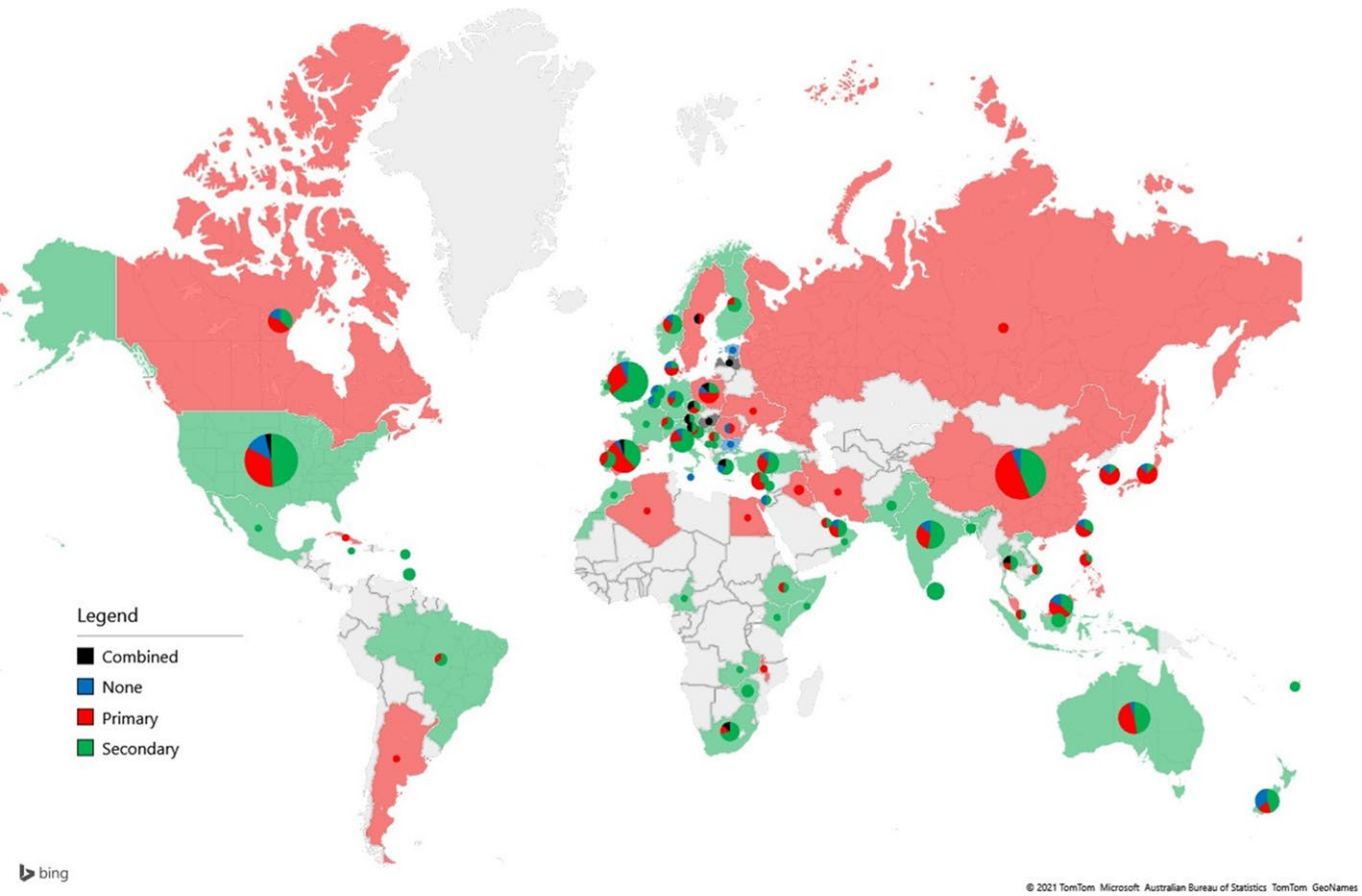
Map of data sources

Figure 5 shows the dominant type of data source used by researchers in each country. The pie charts represent the distribution of the data sources used. Countries are colored based on the most frequent category. In the case of countries where multiple classes have equal frequencies, pie charts are plotted, but the countries are not colorized.

The pandemic situation has resulted in important challenges for tourism researchers regarding what kind of data to work with and how to find valuable input for academic publications. Countries using mostly secondary data for the analyzed academic articles include places with developed (and accessible) statistical data collection, e.g., the UK, Germany, Finland, and Italy, or destinations where the lack of travelers has resulted in a focus on existing data, e.g., African countries. Additionally, in the case of India and Australia, which are two important international tourism markets, the DB included a great number of papers taking advantage of the analysis of existing tourism-related data/information. The research generated by European authors was dominated by the use of secondary information, which may also be motivated by the wide range of available data on European tourism. Places where the primary data collection method was more popular, meaning that more articles in the sample were primary data than using secondary data, include countries that were important source markets of international tourism, such as China or Russia, or areas where actors (policy makers and service providers) were also an important target of academic articles, e.g., Poland and Sweden. The transdisciplinary nature of tourism requires a complex analysis of certain tourism phenomena; however, this is only partly true for the COVID-19-Tourism DB, as most of the countries presented in this DB did not use combined data sources and instead focused on either secondary or primary data sources. Articles using combined secondary and primary data sources mainly originated in Europe, e.g., Hungary, Austria, and Poland. The use of primary or secondary data did not show a strong regional pattern; however, in some cases, only one method was used in the case of the COVID-19-Tourism DB. For example, authors from France, as well as some African and Asian countries, relied on secondary sources, while authors from the Middle East or the Russian Federation had only primary input from COVID-19-related studies. The shift toward using secondary information is a result of a previous trend, where technological advances impact the increasing volume of tourism-related data. In the case of destinations dominated by secondary data, COVID-19 revealed the potential of analyzing already available data sources.

**Fig. 6:**
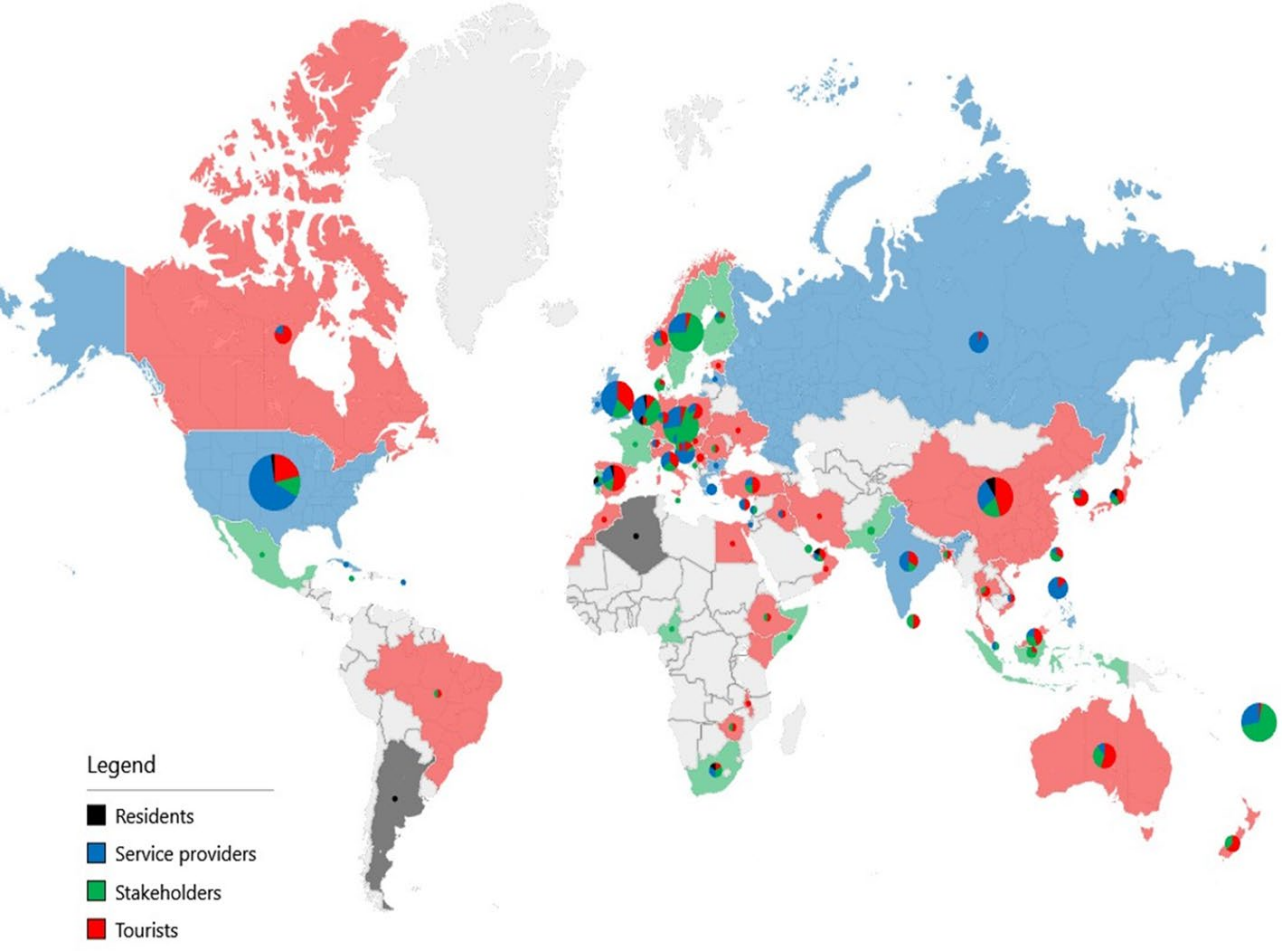
Map of target groups

Figure 6 shows the dominant target groups analyzed by the papers in each country. The pie charts represent the distribution of the analyzed target groups. Please note that the frequency calculated by the target group is not equal to the number of papers since one paper can analyze multiple target groups at the same time. Countries are colored based on the most frequent category. In the case of countries where multiple classes have equal frequencies, pie charts are plotted, but the countries are not colorized.

The impacts of COVID-19 are also reflected in the target groups. The tourist-centric nature of research has become less important, which is parallel with service providers being more addressed by academicians. The target groups addressed in the articles analyzed showed some regional features. Policy makers were more often included in papers originating in Europe, where the DMO system is traditionally more advanced compared with those of other continents or countries. Even in countries where there were more papers addressing tourists (e.g., Germany, Italy, and Norway), the share of policy makers was significant. Tourists are still the most important target group in academic research. In light of COVID-19, the attitudes of tourists, e.g., travel intentions and perception of safety, are at the forefront. Especially in the case of leading source markets in international tourism, such as China or Brazil, the selected works focused on the demand side. Additionally, in the case of emerging markets such as Middle Eastern countries, the demand side was the focus of the selected COVID-19-related academic papers. Moreover, service providers generated a significant volume of academic work in countries where private/business organizations were important and strongly affected by the pandemic (e.g., the US, India or the UK). Local residents were only marginally addressed in COVID-19-related articles. Before 2019, the phenomenon of overtourism induced various studies on tourism’s impact on local communities, and the pandemic situation resulted in a situation in which there was no tourism.

**Fig. 7:**
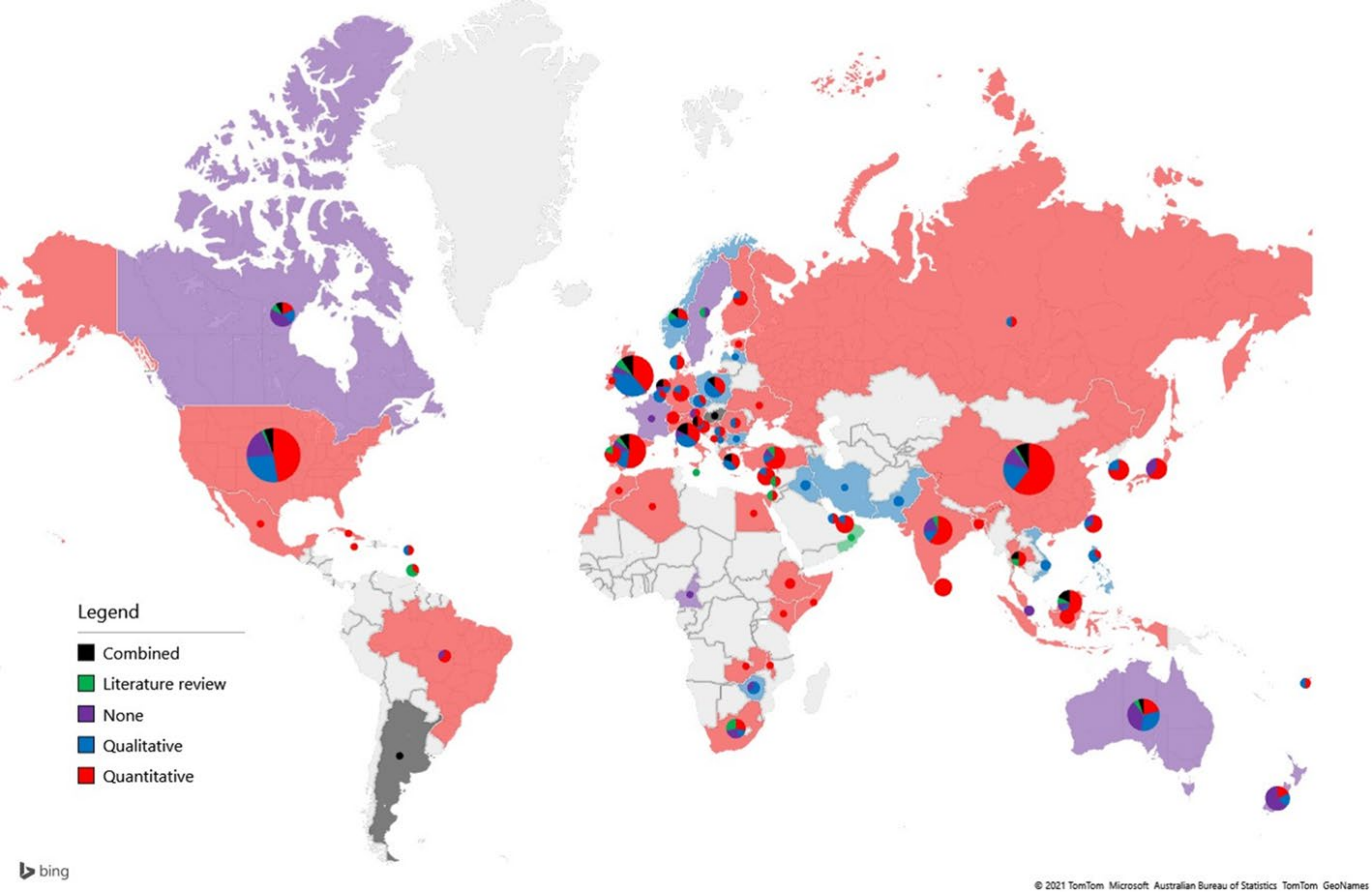
Map of research methods

Figure 7 shows the dominant research method applied by the researchers in each country. The pie charts represent the distribution of papers based on the research method used. Countries are colored based on the most frequent category. In the case of countries where multiple classes have equal frequencies, pie charts are plotted, but the countries are not colorized.

In line with the data source and target groups, the method used showed similar geographical features. Quantitative methods are the most widely used in COVID-19-related articles; in most places/countries, there are papers that have used these methods. However, in some cases, e.g., France, Hungary, Sweden, Iraq, Iran or Pakistan, no quantitative research linked to the pandemic situation was shared. The reasons for the use of the quantitative method may be the objectivity of the results or the comparability or readaption of the same method in other destinations. This use is strongly linked to the availability of data sources in which secondary information has become dominant during the pandemic. The use of such data could have brought about the use of recent quantitative methods , which were previously less used in the field of tourism. The nature of tourism, as a sector, requires a deeper understanding of the system and the mapping of travelers’ viewpoints and behaviors, further supporting service providers and catapulting qualitative research methods to the forefront of tourism research. Literature reviews were found in more advanced economies, e.g., Norway and Sweden, and English-speaking countries, e.g., the US and the UK, or in destinations where the synthesis of existing knowledge may support local actors in rebuilding tourism after COVID-19, e.g., China. In some cases, such as Oman or South Africa, a literature review was an option for entering the international publication landscape. The lower significance of the combined methodology again reflected the strong (and not too wide) focus of the requirements for academic publications.

**Fig. 8:**
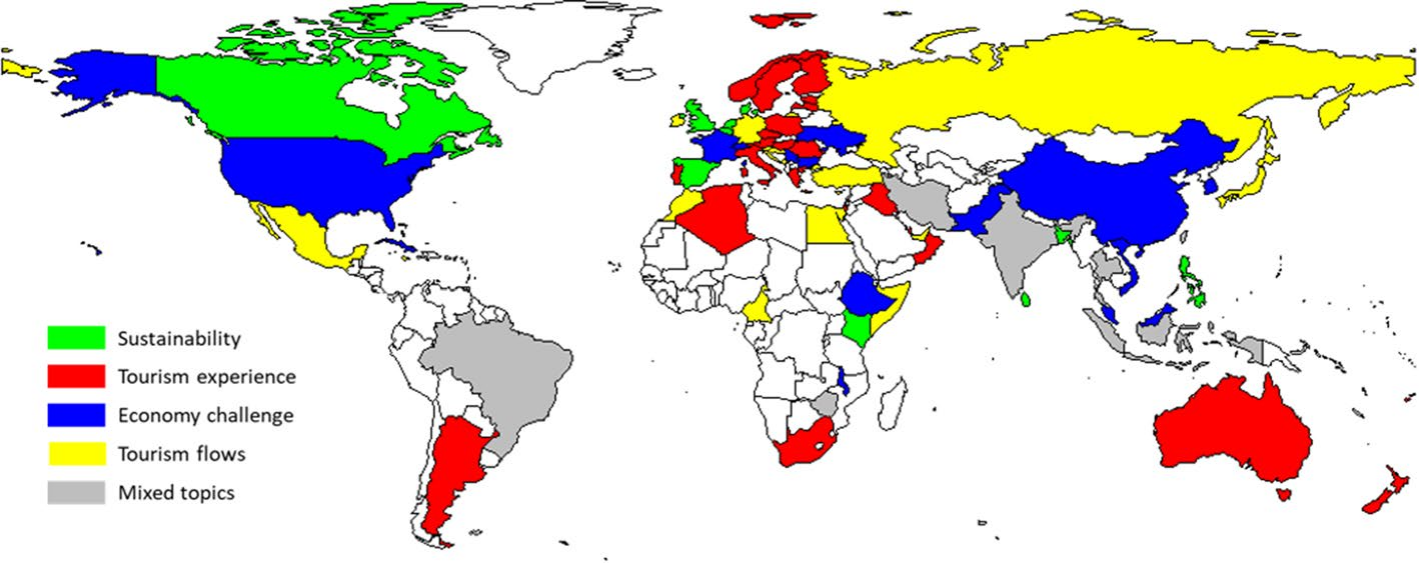
Spatial distribution of dominant topics based on paper abstracts

Figure 8 shows the dominant topic in each country based on paper abstracts. As the initial step, the optimal number of topics was determined by three different metrics provided by Griffiths and Steyvers (2004), Cao et al. (2009), Arun et al. (2010). As suggested by these three metrics, ten topics were selected as the starting model, but similar topics were merged using hierarchical clustering. To measure the similarity between topics, Jaccard similarity was calculated between the sets of the top 20 terms contained by each topic. Finally, five well-defined topics and an additional mixed topic were identified.

Topic modeling resulted in five main research focuses of the academic papers selected from the COVID-19-Tourism DB. In addition to the “mixed” segment, sustainability, tourism experience, economic challenges, and tourism flow were included in research studies focusing on COVID-19-related issues. Topics such as sustainability and economic challenges were also hot topics before COVID-19 and are outlined among future research focuses. Others such as experience or tourism flow have been unveiled by addressing the data sources available during COVID-19. After examining how papers best fit certain topics, tourism experience was found to be dominant, followed by economic challenges. The top papers focusing on the tourism experience dealt with risk perception, which was in line with the pandemic situation. The top articles in terms of economic challenges dealt with the Tokyo Olympics or certain economies, e.g., Greece and Spain. Sustainability issues covered the food sector, social impact or simply a retrospective overview of tourism. In the case of studying tourism flows, mobile phone data analysis was among the top approaches used in the articles. Research on the tourism experience was clearly linked to mature, developed European destinations, such as Scandinavian countries, Italy and Greece from the Mediterranean and Poland or Hungary from Central and Eastern Europe; Australian, South African, and Argentinian authors also dealt with this issue. Regional features could be observed in this case, given Europe’s dominance as the leading continent in terms of international tourism. Although sustainability has recently gained increased attention in the field of tourism, COVID-19 tourism research papers have highlighted its dominance only in some countries that show no regional features, e.g., Canada, Spain, the UK, and Kenya. The COVID-19 pandemic has undoubtedly strongly affected tourism actors, especially profit-oriented companies. The resulting economic challenge, as a topic, emerged and became dominant in destinations with strong private sectors, such as the US or France, or in countries with a strong emphasis on tourism development, such as China. Analyses of tourism flow in light of COVID-19 are fragmented regarding geographical distribution; countries from different continents—e.g., Russia, Germany and Egypt—could provide such a contribution to the academic literature.

**Fig. 9:**
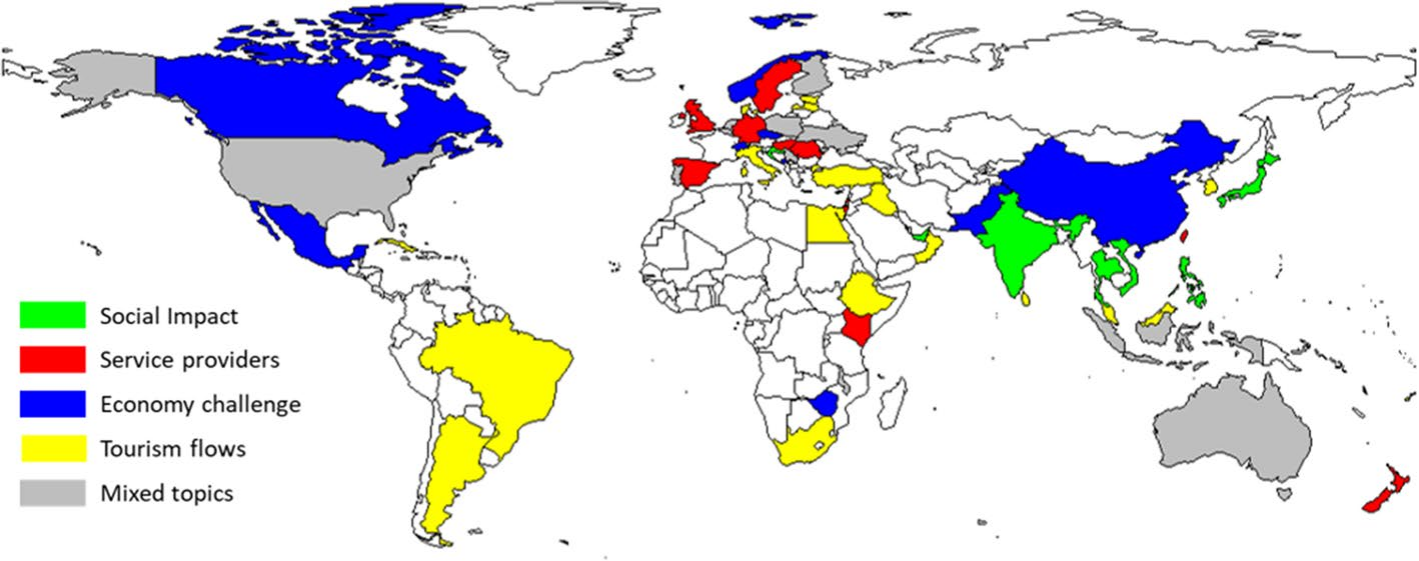
Spatial distribution of the dominant topics based on the research questions of the papers

In the case of topic modeling regarding the research questions, the same approach was used as that described in the case of abstracts. The initial topic number was defined by the metrics suggested by Griffiths and Steyvers (2004), Cao et al. (2009), Arun et al. (2010), which was then refined by applying hierarchical clustering. Six topics were identified, with five well-defined topics and one mixed topic. Figure 9 shows the spatial distribution of the dominant topics. The colors are defined with the consideration of the results obtained through topic modeling conducted on the abstracts. If two topics are similar in content, then they are highlighted with the same color to support better comparability.

Research questions and hypotheses have been included in fewer papers within the COVID-19-Tourism DB, resulting in more “blank” areas on the map. Topic modeling shows some differences when compared with abstracts. In addition to the “mixed” segment, tourism flows and economic challenges comprised a separate segment; however, social impact and service providers formed new groups. Top articles about social impact dominating in Asian countries cover perceptions, attitudes, and influencing factors. The topic of service providers—clearly dominating in European destinations—included recovery strategies and management issues. The economic challenge groups included, among the top articles, those focusing on certain economies, e.g., China, or certain sectors, e.g., Airbnb or food/agriculture. Spatial distribution shows the important role played by service providers in Europe and the role of tourism flows in African, Middle Eastern or Latin American countries.

**Fig. 10:**
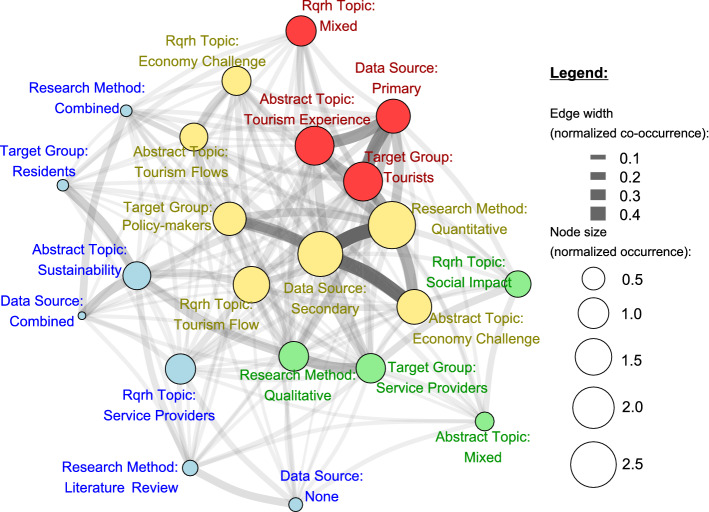
Research network

Figure 10 summarizes the connections among data sources, research methods, target groups, and research topics. These connections specify a *research network*, where the nodes are the kinds of data sources, types of research methods, target groups, topics of abstracts and topics of research questions and hypotheses. The size of the nodes is related to the frequency of the dominant data source (see Fig. 5), the dominant target group (see Fig. 6), the dominant research method (see Fig. 7), the dominant topic of abstracts (see Fig. 8), and the dominant topic of research questions and hypotheses (see Fig. 9) of countries. More precisely, the size of the node is the normalized occurrence, which is the rate of the expected value of its relative frequency.

The edges between nodes represent the co-occurrence of research nodes, and the width of these edges (normalized co-occurrences) is related to the relative frequency of co-occurrence, calculated by Jaccard’s distance. For example, Fig. 10 shows that the secondary data source is primarily evaluated by quantitative methods (22/54) and secondarily by the qualitative method (9/40).

The sizes and co-occurrences of the research nodes are considered mass points, and the applied force atlas algorithm ensures that the largest research nodes (higher occurrences) are organized at the center of the graph. In other words, the center of the research graph specifies the most popular research nodes, such as the most popular kind of data source, the most reviewed research topic, or the most applied research method.

The applied Leiden community detection method provides four groups of research nodes. Since within the community, the edges are denser than between members of two communities, Fig. 10 specifies the typical research profiles.

In the first (yellow) community, the dominant secondary data source was applied, while the typical research method was quantitative. Within this community, the typical target group addressed was policy makers, and the topics were tourism flows and economic challenges. This group had no specific geographical patterns, and authors from different origins dealt with these topics, underlining the “global” effect of the COVID-19 pandemic.

The second (red) group included the primary data source as the dominant input and focused on tourists as the target group and on the tourism experience as the topic. Interestingly, this group had no dominant research methodology; thus, there was a wide range of methods for exploring this topic, probably in line with destination features and information accessibility or data collection options. Similar to the first group, this profile was also globally discussed, with authors originating from many areas, not just one specific region. However, this research profile was typical for one cluster (red color in Fig. 2).

The third (green) group was led by service providers as the target group of the articles, among which academic research was performed mainly via qualitative methods. Here, social impact was the most linked research question; however, the data source may have included both secondary and primary, without one of them being dominant. A wide range of destinations, namely, authors’ affiliations, were included in this group, including leading areas in international tourism, such as the UK, the US and India.

The last (blue) research profile focused on sustainability. The research questions addressed were dominated by service providers’ relevant challenges. Combining data sources and mixed research methodology, literature reviews also belonged to this group. Residents discussed less during COVID-19 as a target group were linked with sustainability. Although the sustainability of the tourism sector is a fundamental issue, this topic had lower relevance for the analyzed COVID-19 tourism articles, so the countries involved (authors’ affiliation) were fewer in number compared with other topics. This research network focused on COVID-19 papers; however, several elements were already present in the mainstream academic research, such as dealing with sustainability and economic challenges. Nevertheless, instead of the tourist-centric focus of the research, more papers dealt with service providers and policy makers. Instead of the qualitative-research focus, recent quantitative methods are employed, not only primary but also secondary data sources. Secondary data sources play the most important role in the research network. It has the highest centrality; therefore, it is organized to the origo. At the same time, secondary data sources limit the possible methods and the research focus.

## Summary and conclusions

Our study that explored the geographical features of selected academic works in tourism resulted in valuable contributions being made to the literature and future directions for tourism research. The present study was designed to determine the effect of the geographical patterns of COVID-19 tourism research. This research was a comprehensive investigation because all of the scientific papers, including the topics “tourism” and “COVID-19” from the Dimensions DB, were collected. Dimensions such as data source, target groups and research methods were categorized, and the geographical dimension was also added to the DB because these dimensions were not integrated into any of the currently available DBs or previous research. The main logic behind this exploratory research was the integration of a systematic literature review with a spatial network. This work contributed to the existing knowledge of academic tourism research by mapping the relationship between the methodological and geographical patterns. This approach will prove useful in expanding our understanding of how geographical and methodological aspects affect academic tourism research. Table 9 provides an overview of the main research results following the research questions, further indicating the most important implications of the current study.

**Table 9:** Summary of research results and implications

Nr.	Research Questions	Results and Contributions	Implications
1	What geographical patterns of tourism research can be identified?	Based on the focus destination, countries can be structured as follows: the countries in one group focus on their own destinations, for example, Brazil and Greece. Another group contains countries focusing on global areas, e.g., the US and Spain. The third group of countries, e.g., Australia and Norway, addresses neighboring problems. In addition, the geographical structure of COVID-19-related tourism studies shows common sociocultural backgrounds, such as those countries in Scandinavia, and direction of tourism flows, such as main destinations, and source markets, such as the US and China. Location is relevant regarding addressed topics and applied methodologies. Regarding paper content, tourism experience has been studied mainly for European countries, while sustainability is a dominant topic in Scandinavian countries and Canada.	This study supports the operation of a worldwide network of tourism academics as the leading knowledge-sharing actors. Furthermore, this study emphasized the significance of cooperation between academics and industry partners. This study shows the potential for regional cooperation among tourism academics representing destinations with similar issues/challenges.
2	Can research profiles and their relations be identified by mapping the methodological aspects of tourism research?	The results show that research profiles can be identified by the proposed method. Applying a multilayer network approach, four different research profiles were identified: 1) the first profile applies secondary data sources and quantitative methods; 2) the second profile relies on primary data sources with the focus of tourists; 3) the third profile is led by service providers being the target group, analyzed by qualitative methods; and 4) in the fourth profile, the topic of sustainability dominates and also includes literature reviews.	Research profiles help scholars measure the link between research characteristics in a specific research field. Using this methodology may be even more important in tourism areas where inductive research dominates; namely, specific observations can be generalized. This study reveals that the application of research profiles is an adequate method through which to examine how scholars react in situations where the global halting of tourism occurs; however, the publishing pressure placed on scholars remains strong. The results of the study may validate tourism research methods and, thus, provide guidance for academic researchers including those in their early tourism career.
3	What effects of COVID-19 on academic tourism research can be identified?	In tourism research, COVID-19 has been shown to affect the research characteristics. During the pandemic, the usage of secondary data sources became dominant. Secondary data include statistical databases and user (traveler)-generated content. The application of quantitative methods has become more frequent in tourism research. Studies focusing on the local community and its link with tourism were less addressed during COVID-19. The concentration of tourism knowledge continued during COVID-19, with leading authors and institutions playing a central role in the dissemination of relevant knowledge and providing guidance during the pandemic.	This study points out that due to the increased usage of secondary data sources and quantitative methods, the development of analytical skills is important for tourism academics in keeping up with publishing pressure.

### Theoretical implications

The significant findings emerging from this study are threefold. First, the geographical patterns of tourism research have been identified ($\hbox {RQ}_1$). Second, certain research profiles have been identified ($\hbox {RQ}_2$). Third, the paper has explored how COVID-19 has impacted academic tourism research ($\hbox {RQ}_3$).

The strong link between geography and tourism is reflected in academic research as well. This study has addressed the geographical patterns on three levels: input (e.g., citation network and destination of focus), methodology and content of the involved articles. The importance of the geographical pattern is influenced by leading academic authors and institutions, the tourism structure and sociocultural background of a certain destination, the development of a statistical data collection system and the current challenges observed in the destination. This study pointed out that for tourism research, geographically related areas have to be investigated by addressing their geographical base. In conclusion, adding the geographical dimension to the analysis of academic research is important in sectors where location is relevant (e.g., commerce, innovation, entrepreneurship, and the circular economy).

The analyzed dimensions generated certain research profiles where the link between methodological components could be measured. Compared with previous studies, Leiden’s community detection obtained unanticipated results, namely, that the element type included in a group may vary (e.g., there is no dominant data source or there are more topics within the same group). Using this methodology to define research profiles may be highly appropriate in tourism areas where inductive research dominates; namely, specific observations can be generalized.

Regarding the effect of COVID-19 on academic tourism research, the current study provides a snapshot of those works that have been covered/included in international scientific journals. Our approach reveals how academics can reflect on a situation where the analyzed phenomenon, i.e., the global halting of tourism, occurs; however, the publishing pressure placed on scholars remains strong. According to our results, there is a shift toward using new (available but not benefited from previously) data sources that require new analytical skills. As a consequence of publishing pressure, a growing number of article types, other than original research articles (e.g., short communication or articles without data input), have been published in international journals.

### Practical implications

Practical implications regarding the geographical pattern have two main pillars. On the one hand, the research explored the potential cooperation among academic tourism researchers that can be based on a certain topic, similar tourism structure of the involved destination or previous cooperation (e.g., coauthorship and organizational contacts). Our study supports the need for a worldwide network among tourism academics who can be leading actors in terms of knowledge sharing. The analyzed phenomenon, namely, COVID-19, is a global issue, and this study has revealed an opportunity for smaller destinations to enter the international academic landscape. On the other hand, the current study has pointed out the significance of cooperation between academics (those who are doing the research) and industry partners (those who provide input, e.g., data for research). Industry partners gain additional benefits because the data are analyzed by professionals, which supports the third mission, i.e., authors’ HEIs.

From a methodological point of view, the current study has revealed the potential of available data sources in tourism (e.g., statistical figures, travelers’ reviews, cell information, company DBs, and websites). These resources were also accessible before the pandemic; however, due to COVID-19 and because of the related limitation of data collection during this period, there has been a shift toward the increased use of this information in tourism academic research. In parallel, the development of analytical skills was also observed, which provides an opportunity for tourism academics to improve their skills through cooperation (e.g., involving colleagues with a high level of analytical skills) or the use of dedicated software (e.g., VOSviewer).

Our study identified some shifts/changes caused by COVID-19 in terms of academic research focus, including the main topics and target segments. Among the main topics discussed in the articles, highly relevant issues, such as sustainability, remained important. Furthermore, COVID-19 resulted in the increased role of addressing tourism flows and highlighted issues such as economic challenges. In some cases, i.e., targeting residents, the current study observed a lower volume of COVID-19-related academic papers.

Taken together, the findings of this study have a number of important implications for restarting tourism and provide inputs for tourism academics to make valuable contributions to the tourism field. Our conclusion is that new tourism needs new approaches in academic tourism research.

## Limitations and future works

Even though COVID-19 has had a global impact on tourism, a limitation of the current study is that the DB contains COVID-19-related articles. Notwithstanding this limitation, the elaborated research design could be used in the case of other tourism issues (e.g., sustainability) to address the role of the geographical dimension.

The scope of this study was limited to tourism, in which the geographical dimension played a role. In the case of other fields, i.e., mathematics or physics, where location was of no importance, there is a limitation to applying this methodology. Because of the lack of former studies on the identification of research profiles, this study followed a data-driven approach instead of a model-driven approach. Since there is no existing theoretical framework for the geographical aspects of research profiles, without any preliminary assumptions, the applied data-driven approach specified the typical research profiles and the relationships between their components. Nevertheless, the result of the data-driven analysis, i.e., a research network that can later be treated as a model, was specified to identify the relationships of the components of research profiles. Future studies should extend this method to include temporal changes to compare pre-COVID-19 and post-COVID-19 research in tourism. Additionally, future studies should indicate how to generalize this method to other regional studies.

Based on our results, we highlight the main challenges of tourism research in the post-COVID-19 period. First, in this paper, the main topics of tourism research, such as tourism experience, tourism flows, sustainability and economic challenges, were revealed. A challenge is to investigate the changes in the importance of these topics in the post-COVID-19 period and how to react to these changes (e.g., support of research projects in certain topics). Second, our paper has identified a shift toward the secondary data source. The usage of secondary data sources insinuates the use of strong methodological approaches and applications in tourism research in the post-COVID-19 period. However, the issue of how to exploit the advantages of the novel methodological approaches in tourism research has arisen as a result.

Opportunities for employing recent methodologies in tourism research raise the question of how this employment can be realized in the future. Our study has not addressed this question but rather unveiled the potential for future research regarding what kind of new skills, cooperation or supporting technological tools can help tourism researchers.

The current study provides a snapshot of academic tourism research; thus, any tendencies or shifts should be elaborated in the future. This research has brought up some issues in need of further investigation. In line with the outcomes of this study, future research could have the following scopes: cooperation among tourism academics and industry partners, knowledge sharing between tourism academics and industry partners, the potential of developing research skills, how to take advantage of existing data sources, and the contribution of the tourism academic field to the development of the tourism sector.

## Supplementary Information

Below is the link to the electronic supplementary material.

Supplementary file 1 (pdf 105 KB)

## Data Availability

The datasets used and/or analyzed during the current study are available from the corresponding author upon reasonable request.

## Footnotes

1 see: https://www.adscientificindex.com.

2 see: https://www.shanghairanking.com.
