# Supplementary material for: Does geography matter? Implications for future tourism research in light of COVID-19
Source: Scientometrics. 2023 Jan 11;128(3):1601–37. doi: 10.1007/s11192-022-04615-z (PMC9833032; doi:10.1007/s11192-022-04615-z)
Supplement: Supplementary file 1 — (pdf 105 KB) [file 11192_2022_4615_MOESM1_ESM.pdf]

Scientometrics manuscript No.
(will be inserted by the editor)

Does geography matter? Implications for future tourism research in light of COVID-19 - Supplementary material

Received: date / Accepted: date

1 Definition of research categories

**Research category**	**Definition**	**Reference**
Quantitative research	"Quantitative research is an approach for testing objective theories by examining the relationship among variables. These variables, in turn, can be measured, typically on instruments, so that numbered data can be analyzed using statistical procedures."	(Creswell, 1994, p. 4)
Qualitative Research	"Qualitative research is an approach for exploring and understanding the meaning individuals or groups ascribe to a social or human problem. The process of research involves emerging questions and procedures, data typically collected in the participant's setting, data analysis inductively building from particulars to general themes, and the researcher making interpretations of the meaning of the data."	(Creswell, 1994, p. 4)
Mixed	Combined or "mixed methods research is an approach to inquiry involving collecting both quantitative and qualitative data, integrating the two forms of data, and using distinct designs that may involve philosophical assumptions and theoretical frameworks."	(Creswell, 1994, p. 4)
None	Neither of them.	

Table 1 Research categories

Address(es) of author(s) should be given

2 Definition of data source types

Data source	Definition	Reference
Primary	"Informaton that researchers gather first hand."	(Rabianski, 2003, p. 43)
Secondary	"Information from secondary sources, i.e. not directly compiled by the analyst;may include published or unpublished work based on research that relies on primary sources of any material other than primary sources used to prepare a written work."	(Rabianski, 2003, p. 43)
Combined	Used both primary and secondary data sources.	
None	Neither of them.	

Table 2 Data sources

3 Definition of target groups (stakeholder)

Stakeholder is defined as "any group or individual who can affect or is affected by the realization of an organization's purpose" (Freeman et al., 2010, p. 26). Within the tourism industry, stakeholder groups defined by Sautter and Leisen (1999) includes: local businesses, residents, activist groups, tourists, national business chains, competitors, government, employees.

Target group	Definition	Reference
Residents	"The residents of a country are individuals whose centre of predominant economic interest is located in its economic territory. For a country, the non-residents are individuals whose centre of predominant economic interest is located outside its economic territory. `https://www.unwto.org/glossary-tourism-terms`"	
Service providers	Following the Sautter and Leisen (1999)'s classification the category includes: local businesses, national business chains, competitors, employees	Sautter and Leisen (1999)
Policy makers	Following the Sautter and Leisen (1999)'s classification the category includes: governments and activist groups	Sautter and Leisen (1999)
Tourists	"Tourist (or overnight visitor): A visitor (domestic, inbound or outbound) is classified as a tourist (or overnight visitor), if his/her trip includes an overnight stay, or as a same-day visitor (or excursionist) otherwise. `https://www.unwto.org/glossary-tourism-terms`"	

Table 3 Target groups

References

Abstract Creswell, J. W. (1994). Research design.

Freeman, R. E., Harrison, J. S., Wicks, A. C., Parmar, B. L., and De Colle, S. (2010). Stakeholder theory: The state of the art.

Rabianski, J. S. (2003). Primary and secondary data: Concepts, concerns, errors, and issues. *The Appraisal Journal*, 71(1):43.

Sautter, E. T. and Leisen, B. (1999). Managing stakeholders a tourism planning model. *Annals of tourism research*, 26(2):312–328.
